# Ulzera und diabetischer Fuß: Moderne und alternative Behandlungsstrategien

**DOI:** 10.1007/s00105-024-05442-4

**Published:** 2024-12-17

**Authors:** Andrei Tanasov, Lawrence Chukwudi Nwabudike, George-Sorin Tiplica

**Affiliations:** 1 Colentina Klinik, Dermatologie 2, „Carol Davila“ Universität für Medizin und Pharmazie, Bukarest, Rumänien; 2 N. Paulescu Nationales Diabetes Institut, Bukarest, Rumänien

**Keywords:** Wundheilung, Plättchenreiches Plasma, Ozontherapie, Stammzellen, Akupunktur, Wound healing, Platelet-rich plasma, Ozone therapy, Stem cells, Acupuncture

## Abstract

Die Komplexität des diabetischen Fußes resultiert aus komplexen pathophysiologischen Mechanismen, verschiedenen klinischen Erscheinungsformen, schweren Komplikationen mit erheblichen Beeinträchtigungen der Lebensqualität und dem Bedarf an speziellen, individualisierten Behandlungen. Insbesondere im Fall von diabetischen Fußgeschwüren sind klassische Therapien nicht immer effektiv, daher werden neue Behandlungsstrategien benötigt. Die vorliegende Übersicht zielt darauf ab, alternative Therapien mit aktuellen Wirksamkeitsdaten für diabetische Fußgeschwüre vorzustellen, die geeignete Optionen bei refraktären Geschwüren sein könnten. Plättchenreiches Plasma und Stammzellen haben eine regenerative und entzündungshemmende Wirkung und zeigen gute Ergebnisse bei der Behandlung von diabetischen Geschwüren, während die Ozontherapie die lokale Durchblutung und den Antioxidationsstatus verbessert. Tragbare Geräte könnten entscheidend für die langfristige Überwachung von Patienten mit diabetischem Fuß werden, da sie die Identifizierung von Geschwüren oder Infektionen frühzeitig ermöglichen. Es gibt auch Literaturberichte über alternative Behandlungen (Madenbehandlung, Honigverbände, Akupunktur), die erfolgreich bei refraktären Geschwüren eingesetzt wurden und aufzeigen, wie vielfältig das Management des diabetischen Fußes sein kann.

## Abstract (en)

Treatment of diabetic foot is challenging due to the complex pathophysiological mechanisms, various clinical manifestations, severe complications with considerable impairment of quality of life, and the need for special, individualized treatment strategies. Particularly in the case of diabetic foot ulcers, conventional therapies are not always effective, so new treatment strategies are needed. This review aims to present alternative therapies with current efficacy data for diabetic foot ulcers that may be suitable for treatment-refractory ulcers. Platelet-rich plasma and stem cells have regenerative and anti-inflammatory effects and show good results in the treatment of diabetic ulcers, while ozone therapy improves local circulation and antioxidant levels. Wearable devices could become crucial for the long-term monitoring of patients with diabetic foot as they allow early identification of ulcers or infections. There are also literature reports of alternative treatments (e.g., maggot debridement, honey dressings, acupuncture) that have been used successfully for treatment-refractory ulcers, demonstrating how diverse the management of the diabetic foot can be.

Die Bezeichnung „diabetischer Fuß“ bezeichnet zusammenfassend Fußerkrankungen bei Diabetikern, die eines oder mehrere der folgenden Merkmale aufweisen: periphere Neuropathie, periphere Arterienerkrankung, Infektion, Hautgeschwür, Neuroosteoarthropathie, Gangrän, Amputation, gemäß der International Working Group on the Diabetic Foot (IWGDF) 2023 [1].

Ein herausragendes Merkmal der Erkrankung ist das diabetische Fußgeschwür, eine schwere Komplikation, die die Sterblichkeit der Patienten erheblich erhöht. Die Pathophysiologie der diabetischen Geschwüre ist komplex und umfasst Polyneuropathien, Arteriopathien, Xerose und sekundäre Infektionen.

Als klassische Behandlung der diabetesbedingten Fußkrankheit gelten die Verringerung der Risikofaktoren, die Behandlung bestehender Läsionen und Störungen (Neuropathie, Arteriopathie, Infektionen), die Vermeidung zukünftiger Verletzungen und die Patientenschulung. Hornhaut und Hyperkeratose sollten entfernt werden, und langfristige Keratolytika sowie feuchtigkeitsspendende Cremes sollten angewendet werden, um Juckreiz zu reduzieren und Kratzverletzungen zu vermeiden, während geeignetes Schuhwerk ausreichend Platz gewährleisten, Deformitäten anpassen und unnötigen Druck vermeiden soll.

Eine gute Blutzuckerkontrolle ist für Neuropathien unerlässlich. Vaskulopathien benötigen möglicherweise Vasodilatatoren und Thrombozytenaggregationshemmer, in schwereren Fällen sogar eine Angioplastie oder Bypassoperation.

Pilz- und Bakterieninfektionen müssen umgehend mit entsprechenden antimikrobiellen Medikamenten behandelt werden, und schwere Fälle wie nekrotisierende Fasziitis könnten eine Amputation erfordern, um das Leben des Patienten zu retten.

Diabetische Fußgeschwüre erfordern besondere Aufmerksamkeit und die konventionellen Behandlungen umfassen hauptsächlich das Wunddébridement (chirurgisch, enzymatisch oder durch Ultraschall), um nekrotisches Gewebe und bakterielle Biofilme zu entfernen, Verbände (Schutzbarrieren, einige auch mit antibakterieller Wirkung), Druckreduzierung, hyperbare Sauerstofftherapie und Vakuumgeräte (die große Mengen Exsudat sammeln können, die Wunde trocken halten und die Sauberkeit sowie die Blutzirkulation verbessern) [2].

Weitere Informationen zur klinischen Präsentation, Diagnose, Klassifikation, zu potenziellen Komplikationen und zum konventionellen therapeutischen Ansatz bei diabetischen Fußkrankheiten finden sich im Kapitel über diabetesbedingte Fußkrankheiten im Buch von Fritz und Tiplica (2024) [3]

In den letzten Jahren wurden neue und vielfältige Behandlungsoptionen mit vielversprechenden Ergebnissen identifiziert, die weitere, umfassendere Studien erfordern, jedoch effiziente therapeutische Optionen für refraktäre diabetische Geschwüre und die diabetische Fußkrankheit im Allgemeinen darstellen könnten. Diese sollen im Folgenden zusammenfassend dargestellt werden.

## Plättchenreiches Plasma

Plättchenreiches Plasma (PRP) ist ein autologes Produkt, das durch Zentrifugation aus peripherem Blut gewonnen wird und hohe Konzentrationen von Blutplättchen sowie plättchenassoziierte Wachstumsfaktoren enthält [4]. PRP wirkt durch die Freisetzung zahlreicher Wachstumsfaktoren im Zielgewebe, die die Regeneration und Gewebereparatur fördern (indem sie auf mehrere Phasen der Wundheilungskaskade einwirken: Entzündung, Proliferation und Remodellierung) und die möglicherweise sogar antibakterielle Eigenschaften sowie schmerzlindernde und immunmodulierende Effekte haben [5].

Diese Prozesse scheinen die Wundheilung zu unterstützen und sogar zu beschleunigen, verglichen mit den konventionellen Therapien: eine Heilungsrate OR (Odds Ratio) = 4,37, 95 %-CI (Konfidenzintervall): 3,02‑6,33, *p* < 0,001; eine durchschnittliche Heilungszeitdifferenz von −3,21 Tagen, 95 %-CI: −3,83 bis −2,59, *p* < 0,001, wie in einer systematischen Übersicht über 20 Studien zu PRP für diabetische Fußgeschwüre berichtet wird [6].

Die durch PRP ausgelösten Prozesse scheinen die Wundheilung zu unterstützen und zu beschleunigen

Die Ergebnisse waren in den Studien unterschiedlich – Heilungsrate der Geschwüre, Zeit bis zur Heilung, Verringerung der Geschwürgröße, Infektionsrate der Wunden, Bildung neuer Blutgefäße, Amputation, Krankenhauskosten und Lebensqualität.

Allerdings reduzierte die PRP-Therapie nicht signifikant den Prozentsatz der Amputationen bei den Patienten. Darüber hinaus hatten in einer Gruppe von 80 Patienten mit nicht reagierenden, nicht heilenden diabetischen Fußgeschwüren die PRP-Injektionen eine Erfolgsquote von 63,7 % nach 4 Wochen (definiert als mehr als 90 % Heilung des Geschwürs) mit signifikant besseren Erfolgsquoten in den Untergruppen mit normalem Body Mass Index (BMI) und kurzer Geschwürdauer. Solche Ergebnisse unterstützen den potenziellen Einsatz von PRP-Therapien für lang anhaltende Geschwüre [7].

Andere Faktoren, die mit dem Erfolg von PRP bei Geschwüren in Verbindung standen, wurden in einer Studie mit 182 Teilnehmern berichtet, die zur Vergleichbarkeit in 2 Gruppen von geheilten vs. nicht geheilten Geschwüren nach der Behandlung unterteilt wurden: Eine längere Dauer des Diabetes mellitus (Mittelwert 17,9 vs. 22,5 Jahre, *p* = 0,003), Raucherprävalenz (39,7 % vs. 58,5 %, *p* = 0,016) oder eine glomeruläre Filtrationsrate unter 60 (44 % vs. 63,4 % der Teilnehmer in der Gruppe, *p* = 0,028) waren signifikant mit schlechteren Ergebnissen assoziiert [8]. Die Metaanalyse von 8 randomisierten kontrollierten Studien, die 598 ältere Patienten mit diabetischen Fußgeschwüren, die mit autologischen PRP-Gelen behandelt wurden, umfasst, zeigt statistisch signifikant bessere Ergebnisse im Vergleich zu konventionellen Therapien in Bezug auf eine kürzere Heilungszeit (Mitteldifferenz −16,97 Tage, 95 %-CI: −32,64 bis −1,29 Tage, *p* < 0,00001) sowie eine kürzere Krankenhausaufenthaltsdauer (Mitteldifferenz −20,11 Tage, 95 %-CI: −38,2 bis −2,20, *p* = 0,03) [9].

Angesichts der Tatsache, dass PRP ein kostengünstiges und leicht zu beschaffendes Produkt ist, sind solche Ergebnisse vielversprechend und können die Behandlung von diabetischen Fußgeschwüren verbessern.

## Stammzelltherapie

Mesenchymale Stammzellen scheinen die Angiogenese und Reepithelialisierung zu fördern und gleichzeitig Entzündungen zu reduzieren – Eigenschaften, die sie zu einem attraktiven Forschungsthema für die Anwendung bei diabetischen Fußgeschwüren machen.

Mesenchymale Stammzellen können aus verschiedenen Geweben, wie z. B. Knochenmark, Fettgewebe und Plazenta, extrahiert werden und wurden sowohl topisch (topische Anwendung, Injektion, Gel) als auch systemisch (z. B. intravenös) bei Patienten mit diabetischen Fußgeschwüren verabreicht [2]. Mesenchymale Stammzellen werden durch Chemotaxis zum geschädigten Gewebe angezogen und differenzieren sich entsprechend den lokalen zellulären Bedürfnissen, wie z. B. der Neovaskularisation, die die Wundheilung beschleunigt [10].

Obwohl sie schwer zu beschaffen sind, wurden mesenchymale Stammzellen bei therapieresistenten diabetischen Geschwüren untersucht, mit Verbesserungen in der Geschwürrückbildung, z. B. mit 59–67 % im Vergleich zur Ausgangsbasis, wenn ABCB5+ mesenchymale Stammzellen als topische Behandlung in einer Studie mit 23 Teilnehmern angewendet wurden. Die Anwendungen wurden gut vertragen, ohne beobachtete unerwünschte Ereignisse [11].

## Ozontherapie

Die topische Ozontherapie basiert auf der Bildung von Peroxiden mit antibakterieller Wirkung sowie auf der Verbesserung der Zirkulation, der Gewebeoxygenierung, des Glukose- und Fettsäuremetabolismus und der antioxidativen Aktivität [12]. Eine Reihe von Studien in den letzten Jahren hat die vielversprechende Wirksamkeit von Ozonbehandlungen bei chronischen diabetischen Geschwüren gezeigt sowie ein gutes Verträglichkeitsprofil; die Daten zu dieser Therapie sind jedoch noch begrenzt.

Eine Metaanalyse von 12 Arbeiten, die topische und systemische Ozonabgabetechniken umfasst, zeigt, dass die Ozontherapie zu einer signifikant schnelleren Verbesserung der Wundfläche sowie zu einem geringeren Amputationsrisiko (RR [relatives Risiko] = 0,36, 95 %-CI: 0,24–0,54, *p* < 0,00001) im Vergleich zur konventionellen Geschwürbehandlung führte. Ein signifikanter Unterschied im Prozentsatz der Patienten mit vollständig geheilten Geschwüren oder in der Dauer des Krankenhausaufenthalts wurde jedoch nicht festgestellt [13].

Eine kurzfristige Verabreichung von Ozon bei 41 Patienten mit diabetischen Fußgeschwüren zeigte ebenfalls signifikant verbesserte Ergebnisse nach 1 Woche im Vergleich zu 48 konventionell behandelten Kontrollen: höhere Superoxiddismutase-Aktivität und gesamte antioxidative Kapazität sowie niedrigere Werte für C‑reaktives Protein, Interleukin‑6, Erythrozytensedimentationsrate und TNF(Tumornekrosefaktor)-α (*p* < 0,05); die Wirkungen der Ozontherapie hielten langfristig an und resultierten in kürzeren Krankenhausaufenthalten [14]. Die Ozontherapie scheint auch die Schmerzsymptome zu lindern, wie bei 54 Patienten mit diabetischen Fußgeschwüren berichtet wurde, die mit der visuellen Analogskala bewertet wurden: eine Reduktion von einem Median von 6 (5 bis 7) Punkten auf 4,4 (3 bis 7) Punkte nach einem Zyklus der Ozontherapie (*p* = 0,000001) [15].

## Hyperbare Sauerstofftherapie

Dabei handelt es sich um eine bereits bekannte und angewandte Therapie für diabetische Fußgeschwüre, die nachweislich Wirksamkeit zeigt, die vollständige Heilungsrate erhöht und die Amputationsrate senkt im Vergleich zur konventionellen Behandlung [16].

Ein Vergleich zwischen Ozon- und hyperbarer Sauerstofftherapie in einer Studie mit 114 Patienten mit venösen Beingeschwüren, die 30 Zyklen der jeweiligen Therapie durchliefen, zeigt, dass beide Therapien die Wundfläche signifikant reduzierten (um 69,67 ± 22,52 % in der hyperbaren Sauerstoffgruppe vs. 41,33 ± 21,31 % in der Ozongruppe), jedoch mit besseren Ergebnissen für die hyperbare Sauerstofftherapie [17]. Dennoch sind weitere Studien an größeren Teilnehmergruppen und speziell zu diabetischen Geschwüren erforderlich.

## Einlagen

Moderne Systeme mit Einlagen, die eine kontinuierliche Überwachung und Rückmeldung verschiedener Parameter gewährleisten, könnten einen wichtigen Ansatz zur Prävention von diabetischen Fußkomplikationen darstellen. Beispielsweise berichtete eine randomisierte kontrollierte Studie (RCT) mit 46 Patienten mit diabetischer peripherer Neuropathie, dass die Gruppe, die ein intelligentes Einlegesohlensystem trug, das Audio-, visuelle und vibrationale Warnungen ausgab, wenn ein zu hoher plantarer Druck festgestellt wurde, eine Reduktion der Geschwürinzidenz um 71 % erreichte [18]. Die Druckentlastung ist entscheidend zur Verhinderung diabetischer Geschwüre, und ein solcher Sensor ermöglicht es den neuropathischen Patienten zu erkennen, wann der plantare Druck alarmierende Werte erreicht hat. Fußtemperaturüberwachungssysteme haben ebenfalls einen positiven Einfluss auf die Prävention von diabetischen Fußkomplikationen gezeigt: Der tägliche Fußtemperaturscan mit einem solchen Temperaturüberwachungsgerät hatte eine signifikante Genauigkeit von 97 % bei der Vorhersage der Bildung eines diabetischen Geschwürs, basierend auf Temperaturasymmetrien [19]. Ähnliche Temperatursensoren wurden erfolgreich zur Identifizierung diabetischer Fußinfektionen eingesetzt [20]. Einlagen, wie die bereits erwähnten, werden zunehmend personalisierter, basierend auf den anatomischen Besonderheiten des Fußes der Patienten, unter Verwendung von 3‑D-Druckern [21], oder sogar in Form von Socken, die kontinuierlich die Temperaturen des diabetischen Fußes überwachen und die Daten an Patienten übermitteln. Sie warnen, wenn ein Temperaturunterschied festgestellt wird, um ein potenzielles Geschwür rechtzeitig zu erkennen [22]. Solche Socken wurden von den Teilnehmern der Studie gut vertragen, die keine Unterschiede zu ihren üblichen Socken angaben. Tragbare Sensoren können auch verwendet werden, um posturale Veränderungen zu bewerten, die signifikant mit tiefer diabetischer Neuropathie (*p* < 0,05) und einer schlechten BZ(Blutzucker)-Kontrolle (*p* < 0,05) korreliert sind, wie in einer Studie mit 85 Patienten berichtet wurde, die aufgrund von Gleichgewichtsstörungen sturzgefährdet waren [23].

## Akupunktur und Moxibustion

Evidenzbasierte Berichte über den Erfolg traditioneller chinesischer Medizin bei der Behandlung des diabetischen Fußes wurden kürzlich veröffentlicht. Eine Pilotstudie mit 18 Patienten mit nicht heilenden chronischen Fußgeschwüren, verursacht durch Diabetes oder periphere arterielle Erkrankungen, bewertete mehrere Mikrozirkulationsparameter wie die Sauerstoffsättigung des Hämoglobins, den relativen Blutfluss und die Blutflussgeschwindigkeit nach einer 5‑minütigen Sitzung von Akupunktur zwischen den Akupunkturpunkten Magen 14 und 15 (die sich in den ersten beiden Interkostalräumen befinden). Das Ergebnis war eine statistisch signifikante Verbesserung in 7 von 8 mikrozirkulatorischen Parametern, die oberflächlich und tief an den Wundrändern gemessen wurden [24].

Eine Metaanalyse von 12 randomisierten kontrollierten Studien (RCTs), die 1196 Patienten mit diabetischen Fußgeschwüren einbezog, zeigt, dass die Hinzufügung von Moxibustion (Verbrennung von Beifußblättern an bestimmten Körperpunkten) im Vergleich zu den konventionellen Behandlungen zu einer signifikant verbesserten Wundheilungseffizienz (RR = 1,16; 95 %-CI: 1,11–1,22) oder zu einer verkürzten Heilungszeit (mittlere Differenz von −6,27 Tagen; 95 %-CI: −8,68 bis −3,86 Tage) führt [25].

## Alternative topische Behandlungen

Eine retrospektive, vergleichende Studie untersuchte bei 18 Patienten mit 20 Geschwüren Madentherapie gegen konventionelle Methoden bei nekrotischen Geschwüren [26]. Zu Beginn hatte die Madentherapiegruppe eine größere initiale Oberfläche nekrotischen Gewebes (5,0 [2,0–8,0] vs. 5,0 [2,0–8,0] cm^2^) und einen niedrigeren Anteil an Wundabdeckung mit nekrotischem Gewebe (38 % [22–55 %] vs. 44 % [22–67 %]). Der Prozentsatz des vollständigen Wundverschlusses war in der Madentherapiegruppe größer (44 % [22–67 %] vs. 21 % [0–44 %]), mit einer niedrigeren durchschnittlichen Zeit bis zum Wundverschluss (15 [3 bis 26] vs. 18 [8 bis 28] Tage). Dies deutet auf eine Überlegenheit der Madentherapie hinsichtlich Wunddébridement und Heilung im Vergleich zur konventionellen Therapie hin.

Madentherapie ist hinsichtlich Wunddébridement und Heilung der konventionellen Therapie überlegen

Honigverbände (HD) zeigten in einer Metaanalyse von 882 Patienten (424 HD und 458 Kontrolle) eine höhere Wundheilungsrate (OR = 2,06; 95 %-CI: 1,45–2,93, *p* < 0,001) und eine kürzere Wundheilungszeit (MD von −10,42; 95 %-CI: −16,27 bis −4,58, *p* < 0,001) im Vergleich zu den Kontrollen [27]. Eine Einschränkung dieser Metaanalyse war die kleine Stichprobengröße der ausgewählten Studien.

Wir berichteten von einem Fall mit einem langjährigen arteriopathischen Geschwür, der zur Amputation vorgesehen war und erfolgreich mit einer magistralen Zubereitung unter Verwendung der sog. Miculicz-Salbe (die Perubalsam enthält) behandelt wurde [28]. Die Wunde des Patienten heilte ohne Nebenwirkungen, insbesondere ohne allergische Reaktion gegen das als Typ-4-Allergen bekannte Perubalsam.

## Schlussfolgerung

Mit den technologischen Entwicklungen entstehen neue Anwendungen für den medizinischen Einsatz, insbesondere im Bereich der Wundheilung und des diabetischen Fußes. Dieser Zustand erfordert erhebliche Aufmerksamkeit, ständige Pflege, eine rasche Erkennung von Anomalien, die Bewertung des Gesundheitszustands des Patienten und eine angemessene Entscheidungsfindung bezüglich der Notwendigkeit eines Arztbesuchs. All dies kann durch neue Medizintechnologien im Rahmen eines „Hospital-at-Home“-Versorgungsmodells unterstützt werden. Besonders dann, wenn konventionelle Therapien nicht zum Erfolg führen, wie es beim diabetischen Fuß leider häufig der Fall ist, können alternative Behandlungen in Betracht gezogen werden, die manchmal eine überraschende Wirksamkeit aufweisen.

## Fazit für die Praxis

- Bei diabetischen Fußgeschwüren sind klassische Therapien nicht immer effektiv.
- Alternative Therapien für diabetische Fußgeschwüre wie plättchenreiches Plasma, Stammzellen, Ozontherapie, hyperbare Sauerstofftherapie und tragbare Geräte für die langfristige Überwachung von Patienten mit diabetischem Fuß können eine geeignete Option bei refraktären Geschwüren sein.
- Literaturberichte zeigen, dass auch alternative Behandlungen wie Madenbehandlung, Honigverbände, Akupunktur und Moxibustion erfolgreich bei refraktären Geschwüren eingesetzt wurden.
